# Implementation of a novel synchronous multi-site all day high-fidelity simulation

**DOI:** 10.1186/s41077-018-0063-8

**Published:** 2018-02-13

**Authors:** Paul Abraham, Franck Verdonk, Clement Buleon, Antoine Tesniere, Marc Lilot

**Affiliations:** 1 Youth Committee of the French Society in Anesthesia and Intensive Care Medicine (SFAR), Paris, France; 2 Centre Lyonnais d’Enseignement par la Simulation en Santé (CLESS), SAMSEI, Claude Bernard University Lyon 1, Health Services and Performance Research Lab (HESPER EA 7425), Department of Anesthesiology and Critical Care medicine, Hospices Civils de Lyon, Lyon, France; 3 Department of Anesthesiology, Critical Care, SMUR, and Burn Unit, GH Saint-Louis-Lariboisière-Fernand Widal University Hospitals, Assistance Publique, Hôpitaux de Paris, Paris, France; 4 Medical Simulation Center Normandie Simulation en Santé (NorSimS), Université Normandie-Caen, Caen University Hospital, Caen, France; 5 Surgical Intensive Care Unit, Cochin Hospital, iLumens Simulation Department, Sorbonne Paris Cité University, Paris, France

**Keywords:** High-fidelity simulation training

## Abstract

Integration of simulation in educational curricula for anesthesia and intensive care residents is a hot topic. There is a great interest for simulation centers to share their experiences through multi-site synchronous simulation sessions. The present study results from an experience conducted at three sites in France (Paris, Lyon, and Caen), which involved 16 instructors and 25 residents facing the same scenario across 1 day. Synchronous simulations were performed at each site with local and shared debriefing via teleconference. This innovative approach to simulation was found to be feasible, although certain difficulties were encountered with connectivity.

## Introduction

There is a growing willingness, both nationally and internationally, for simulation program implementation in curricula of anesthesia and intensive care residents. This is associated with effectiveness in improving performance, patient outcomes, and team management [1–3]. Clear recommendations for the implementation of a simulation program, including financial, human resources, pedagogical, deontological and quality management demands, and infrastructure and equipment requirements, have been published in 2012 by the French health authorities [4]. These have been used by simulation centers across the country, and it is now of interest to share their experiences through a multi-site synchronous simulation session. Shared tele-debriefing using videoconferencing technology was adapted, as has been reported for high-fidelity simulation (HFS) [5]. Although simultaneous simulation sessions between a center performing HFS and a center visualizing it simultaneously for a shared debriefing, or between two centers performing HFS alternatively with a shared trans-Atlantic debriefing [6] have been reported, synchronous multicenter simulation over a whole day has yet to be described.

## Method

This multi-site multi-modality simulation was performed on April 11, 2017, in France between Lyon (CLESS, Université Claude Bernard Lyon 1), Caen (NorSimS Simulation center, Caen University Hospital), and Paris (Ilumens Simulation Department, Sorbonne Paris Cité University).

Twenty-five anesthesiology and intensive care medicine participated in the session requiring 16 instructors and 3 technicians. All residents participated on a voluntary basis. The simulation was organized by all three centers with the support of the French Society in Anesthesia and Intensive Care Medicine (SFAR) youth committee.

Among the Internet protocol-based teleconference software available, such as Polycom™, Skype™, or GoTo Meeting™, we selected the latter since it provides better quality audio and video. Each center had a broadband internet connection, and the connectivity was tested the day prior to the simulation session by center technicians. During the trial session, a full-scale test of all connections and equipment, using exactly the same materials that were to be used during the experience, was performed. The three centers had already standardized the HFS timeline following the French health authority recommendations [4]. This consisted of a first general HFS briefing with participant orientation of the simulator in each center—a short 10-min introductory course to recall all the clinical examination specificities of the HFS mannequin (SimMan 3G high-fidelity mannequin, Laerdal™, Stavanger, Norway), used for the session (one in each center). For each consecutive scenario, the introduction was followed by a specific briefing and the facilitated scenario, a structured debriefing with a descriptive phase, an analysis phase, and finally, a specific synthesis. Each center had more than 3 years of experience in repetitive HFS scheduled for residents. There was continuity between the scenarios throughout the day; the same “patient” was used for each scenario that illustrated different critical situations. Further, the scenarios were developed jointly, and each center had the necessary resources for the implementation of the simulation day.

The scenarios chosen were HFS in pre-hospital emergency settings with a polytrauma patient—the learning objectives were first sight evaluation of severity, team leadership, prioritization of management, airway management in a suspected spine-injured trauma patient, and organization of patient transport and transfer to trauma center; HFS during the first 20 min of admission to the trauma center with intra-abdominal hemorrhagic shock—the learning objectives were information transmission from pre-hospital emergency team to the trauma center team, team leadership, prioritization of management, hemorrhagic shock management, close loop communication and call out, focus assessment sonography, and shared decision-making with surgical team for patient orientation (operating room or CT scan); HFS in the operating room with tension pneumothorax before exploratory damage-control laparotomy—the learning objectives were tension pneumothorax diagnosis and management, prioritization of vital complication management versus surgical procedure, management of leadership with surgical team, and performance of thoracic decompression and drainage; conversational simulation for family announcement of an encephalic dead state—the learning objectives were initial contact with the family, inform the family members about the encephalic death state, deal with the grief reaction, and facilitate this.

Each scenario involved two to four residents, who were debriefed by their “home” instructors and peers for a planned 20- to 30-min period. Residents and instructors from the other centers also actively participated in the discussion through shared debriefings.

The debriefing setting was established, in each simulation center, with the aim to facilitate cooperative and collaborative communication between instructors and residents. The interconnectivity via videoconference enabled simulation center interactions several times during the learning session; the connection between centers was scheduled during the briefing and debriefing periods in the morning and the afternoon, to commonly share feedback on the scenarios and relevant clinical points in the context of local differences.

Evaluation of the day from both learners and simulation managers was performed thanks to an online survey 1 week after the simulation session, in order to capture participant feedback (overall satisfaction and organization of this day), their appreciation of each scenario, and their thoughts regarding the synchronous multi-site aspect of this event.

## Results

No medical performance data was collected. Each of the four scenarios was of approximately 60 min in duration, including a 5-min situational briefing and 20-min simulation scenario immediately followed by 20 to 30 min of debriefing. Shared debriefing lasted between 20 to 30 min, each center reporting on the specific educational experience and interesting topics of discussions that occurred during local debriefing. All centers succeeded in performing all scenarios, even though the timing for each simulation session or debriefing was slightly different between centers.

The videoconference quality was suboptimal across the day (video lag and sound stuttering). Sound distortion also occurred several times, and the connection was disrupted twice during shared debriefing. This disruption hindered the Caen simulation center then the Paris simulation center, to be part of the shared debriefing for the morning and afternoon sessions, respectively.

During the debriefing, only minor management differences arose. Common agreement between centers was found in the debriefing of most scenarios. Hereafter are several examples resulting from the shared debriefing for the trauma center admission scenario. Each center agreed on the need for the team to respect a “no-touch” period in order to follow closely the transmission of relevant clinical information between the pre-hospital physician and trauma center team leader. For this scenario, in particular, the role of residents and the tools for effective team communication during crisis management was recalled, with centers giving feedback on their local experience and practice. Further, the recent literature for the early initiation of vasopressors in hemorrhagic shock and the prioritization of care in the context of a multi-trauma patient was also emphasized.

Six residents completed the feedback questionnaire, but all were very positive about this collaborative simulation session. Two emphasized that video and sound quality of teleconference had to be improved.

## Discussion

This synchronous one-day multi-site simulation was found to be feasible. Furthermore, sharing debriefing with other centers provided insightful comments and thoughts on simulated scenarios. This approach may also become a valuable tool for simulation centers to benefit from the experience of external experts in specific fields of critical care. However, there were certain aspects that require attention in the future, most notably the quality of the videoconference which was suboptimal and impeded the participation of all centers on two occasions. This may be related to the use of free internet teleconference software, and therefore, it may be of interest to consider using professional services for future sessions.

Informal feedback from the residents was very positive, and all were willing to participate in this kind of workshop in the future. The response rate to our survey was low and likely impacted by the 1-week delay in collection.

This collaborative work paves the way for simulation centers to start sharing with others their experiences, expertise, scenarios, resources, and research projects. Such networking between French simulation centers may be extended to an international network and could promote exchanges in terms of practice and organizational skills between different countries. Cooperation, with sharing of human and material resources for a common project, may benefit the present and future generations of intensive care and anesthesiology caregivers thanks to improved simulation-based learning sessions.

## Abbreviation

HFS: High fidelity simulation
